# Supplementary material for: Role of occupational therapy in reducing and managing violence among mental health inpatients: a scoping review protocol
Source: BMJ Open. 2021 Jul 28;11(7):e046260. doi: 10.1136/bmjopen-2020-046260 (PMC8319987; doi:10.1136/bmjopen-2020-046260)
Supplement: Supplementary data [file bmjopen-2020-046260supp001.pdf]

Appendix One – Literature Search Strategy for CINAHL Plus

Search	Date	Query	Result
S4	05/06/2021	S1 and S2 and S3	184
S3	05/06/2021	occupational therap* OR occupational scien* OR occupational performance OR occupational participation OR activity analysis OR occupational identity OR occupational alienation OR occupational deprivation OR occupational disruption OR occupational dysfunction OR occupational imbalance OR occupational adaptation OR occupational *justice OR occupational engagement OR occupationa* focus* OR occupational perspective OR occupational profile OR Model of Human Occupation OR MOHO OR Canadian Model of Occupational Performance and Engagement OR CMOP-E OR Person?Environment?Occupation?Performance model OR PEOP OR Vona du Toit Model of Creative Ability OR VdTMoCA OR Kawa OR Person?environment?occupation OR PEO model OR dark occupation OR sensory OR vocational rehabilitation	100,300
S2	05/06/2021	aggressi* OR violen* OR agitat* OR hostil* OR irritab* OR assault* OR seclusion OR restraint OR offending OR recidivism	140,970
S1	05/06/2021	mental health OR psychiatry OR mental illness OR psychiatric illness OR mental disorder OR psychiatric disorder OR psychopath* OR personality disorder	255,519
