# Supplementary material for: Role of occupational therapy in reducing and managing violence among mental health inpatients: a scoping review protocol
Source: BMJ Open. 2021 Jul 28;11(7):e046260. doi: 10.1136/bmjopen-2020-046260 (PMC8319987; doi:10.1136/bmjopen-2020-046260)
Supplement: Author's manuscript [file bmjopen-2020-046260.draft_revisions.pdf]

BMJ Open

BMJ Open is committed to open peer review. As part of this commitment we make the peer review history of every article we publish publicly available.

When an article is published we post the peer reviewers’ comments and the authors’ responses online. We also post the versions of the paper that were used during peer review. These are the versions that the peer review comments apply to.

The versions of the paper that follow are the versions that were submitted during the peer review process. They are not the versions of record or the final published versions. They should not be cited or distributed as the published version of this manuscript.

BMJ Open is an open access journal and the full, final, typeset and author-corrected version of record of the manuscript is available on our site with no access controls, subscription charges or pay-per-view fees (http://bmjopen.bmj.com).

If you have any questions on BMJ Open’s open peer review process please email info.bmjopen@bmj.com

BMJ Open

The Role of Occupational Therapy in Reducing and Managing Violence amongst Mental Health Inpatients: A Scoping Review Protocol

Journal:	*BMJ Open*
Manuscript ID	bmjopen-2020-046260
Article Type:	Protocol
Date Submitted by the Author:	24-Oct-2020
Complete List of Authors:	Bell, David; University of Birmingham; St Andrew's Healthcare, Occupational Therapy Department<br>Hallett, Nutmeg; University of Birmingham , School of Nursing
Keywords:	PSYCHIATRY, MENTAL HEALTH, FORENSIC MEDICINE

SCHOLARONE™
Manuscripts

I, the Submitting Author has the right to grant and does grant on behalf of all authors of the Work (as defined in the below author licence), an exclusive licence and/or a non-exclusive licence for contributions from authors who are: i) UK Crown employees; ii) where BMJ has agreed a CC-BY licence shall apply, and/or iii) in accordance with the terms applicable for US Federal Government officers or employees acting as part of their official duties; on a worldwide, perpetual, irrevocable, royalty-free basis to BMJ Publishing Group Ltd ("BMJ") its licensees and where the relevant Journal is co-owned by BMJ to the co-owners of the Journal, to publish the Work in this journal and any other BMJ products and to exploit all rights, as set out in our licence.

The Submitting Author accepts and understands that any supply made under these terms is made by BMJ to the Submitting Author unless you are acting as an employee on behalf of your employer or a postgraduate student of an affiliated institution which is paying any applicable article publishing charge ("APC") for Open Access articles. Where the Submitting Author wishes to make the Work available on an Open Access basis (and intends to pay the relevant APC), the terms of reuse of such Open Access shall be governed by a Creative Commons licence – details of these licences and which Creative Commons licence will apply to this Work are set out in our licence referred to above.

Other than as permitted in any relevant BMJ Author's Self Archiving Policies, I confirm this Work has not been accepted for publication elsewhere, is not being considered for publication elsewhere and does not duplicate material already published. I confirm all authors consent to publication of this Work and authorise the granting of this licence.

For peer review only

The Role of Occupational Therapy in Reducing and Managing Violence amongst Mental Health Inpatients: A Scoping Review Protocol

Corresponding author:

David Bell, Occupational Therapist, St Andrew's Healthcare, 70 Dogpool Lane, Birmingham. United Kingdom. B30 2XR dsbell@standrew.co.uk

AND

Health Education England and National Institute for Health Research Integrated Clinical Academic Intern

ORCID ID: https://orcid.org/0000-0002-8693-0537

Co-author:

Nutmeg Hallett, PhD, Lecturer in Mental Health Nursing, University of Birmingham, The School of Nursing, College of Medical and Dental Sciences, Edgbaston, Birmingham. United Kingdom. B15 2TT.

ORCID ID: https://orcid.org/0000-0003-3115-8831

Word count:

3633

For peer review only - http://bmjopen.bmj.com/site/about/guidelines.xhtml

ABSTRACT

Introduction: Violence is pervasive amongst psychiatric in-patients and has profound consequences for its victims, its perpetrators and mental health services. Currently, the unique contribution of occupational therapists to reducing and managing violence amongst this patient group has not been systematically explored. However, an *a priori* model which provides an initial understanding of its role in this respect can be identified from the wider scholarly literature. This scoping review aims to apply this model, and to refine and validate it, thereby producing an amended model that will form the basis for further research.

Methods and analysis: This scoping review is based upon guidance from the Joanna Briggs Institute, Levac, Colquhoun and O'Brien's scoping review framework, and the Preferred Reporting Items for Systematic Reviews and Meta-Analyses Scoping Review checklist. Electronic databases (CINAHL Plus, PsycINFO, MEDLINE, PsycARTICLES, ProQuest Health and Medicine, AMED and Google Scholar) and grey literature will be searched to identify relevant papers. Included articles will apply occupational therapy theory or occupational science to the reduction or management of violence among psychiatric in-patients and will be critically appraised by two independent reviewers. Study characteristics will be presented using frequency counts, and qualitative data will be analysed using 'best-fit' framework synthesis and secondary thematic analysis to produce an overall model of occupational therapy's contribution to violence management and reduction. A final consultation phase involving mental health inpatients, their families and multi-disciplinary mental health professionals will refine and validate the proposed model.

Ethics and dissemination: results will be disseminated through a peer-reviewed academic journal and through the consultation phase of the review. This consultation phase has been designed to incorporate patient and public involvement. Ethical approval will be sought for this stage, however the review prior to this stage will collect secondary data and therefore will not require such approval.

STRENGTHS AND LIMITATIONS OF THIS STUDY

- This study is the first attempt to map out the unique role of occupational therapy in reducing and managing violence in psychiatric in-patient settings.
- Using a combination of 'best-fit' framework synthesis and secondary thematic analysis will provide a model that organises and makes explicit existing knowledge in this respect.
- The consultation phase will ensure the model is shaped by the lived experience of mental health inpatients and their families, and the insights of a variety of mental health professionals.
- Excluding self-harm as an act of violence may result in relevant studies being omitted from the final review.

KEYWORDS

Occupational therapy, occupational science, violence, aggression, mental health inpatients, best-fit framework synthesis

For peer review only - http://bmjopen.bmj.com/site/about/guidelines.xhtml

INTRODUCTION

Violence may be defined as 'an act [committed] with some degree of wilfulness that caused/has the potential to cause physical or serious psychological harm to another person/persons'[1]. Within mental health inpatient settings, violent acts are both chronic and pervasive. Indeed, a recent systematic review of patient safety within these settings reported that 43% of participants (staff and patients) had suffered physical violence and 57% verbal aggression[2].

Whilst evidence from systematic reviews suggests that psychiatric inpatients who commit acts of aggression and violence are most likely to be younger, male, and involuntarily admitted with a diagnosis of schizophrenia[3-4], other studies report high rates of violence amongst entire psychiatric populations. For example, Iozzino et *al's.*[5] meta-analysis reported that nearly 20% of psychiatric acute inpatients display acts of physical violence during their treatment in hospital. Similarly, Broderick et *al.*[6] reported that 31% of patients in a large, multihospital forensic system committed at least one physically violent assault during their hospitalisation. Furthermore, additional studies have shown that violence is a common feature of psychiatric inpatient settings, irrespective of country[2, 5, 7], patients' gender[7-8], patients' diagnosis[7-8] and civil or forensic status[2, 8]. Thus, whilst psychiatric inpatients who act aggressively and violently may commonly fit a particular demographic, violence is by no means the preserve of a small group of psychiatric inpatients.

Given its prevalence, violence in psychiatric inpatient settings inevitably has severe and far-reaching consequences. This is most obviously the case for its victims, who have reported increased physical health problems, decreased emotional well-being and various psychological impairments, most commonly symptoms of post-traumatic stress disorder[9]. Whilst healthcare professionals and patients can both be affected directly in this manner, patients experience further suffering through the reduction in the quality of care they receive from assaulted healthcare workers[9].

Additionally, the perpetrators of violence experience negative outcomes. A recent systematic review concluded that there is some evidence to suggest that agitated and aggressive psychiatric in-patients experience longer periods of admission, increased probability of readmission and increased medication usage[10]. Moreover, they are likely to be affected by restrictive practices, for example seclusion, or chemical or physical restraint, which are used to contain violence and aggression. These interventions are associated with post-traumatic stress disorder in 25%-47% of cases, as well as predominantly negative emotions, including distress and feeling punished[11].

Finally, violence has negative consequences for healthcare services. Practices to contain violence entail significant financial costs, calculated at 822€ per episode[12]. Additionally, patient aggression has been correlated with burnout in forensic psychiatric nurses[13], higher levels of work stress amongst forensic nurses[14], and decreased job satisfaction among healthcare workers[9]. Such factors inevitably increase the financial burden on services, through reduced effectiveness and retention of staff.

The prevalence and impact of violence amongst psychiatric inpatients make it unsurprising that considerable efforts have been made to understand its causal factors. Historically, the dominance of the medical approach within healthcare services has meant that violence has been understood as having pathological origins[15], with its roots in patients' mental illness and personality structures. In contrast, more recent understandings have emphasised the importance of environmental factors and more specifically, their complex interactions with these patient-related factors[15-16]. Thus, recent efforts to understand violence in psychiatric settings have been far more diverse in their scope, and have sought to identify how violence is influenced by (amongst other factors), the physical

For peer review only - http://bmjopen.bmj.com/site/about/guidelines.xhtml

environments of wards[17], nurse and patient interpersonal styles[18], ward social climate and sense of community[19], and coercive measures[15]. Perhaps most comprehensive in this respect is Cutcliffe's and Riahi's[15-16] systemic model of variables that contribute to aggression and violence, which incorporates phenomena related to the environment, individual patients, individual clinicians and a mental health-care system in its entirety. Similarly, Nijman[20] presents a model that identifies patient, ward and environmental factors that can cause violence, however this model outlines more explicitly the interplay between these factors, and the 'vicious circle' whereby they interact not only to cause violence, but to reinforce it. Both of these models highlight the shift away from using a medical approach to understand violence, towards using a biopsychosocial approach.

Guidelines from the National Institute for Health and Care Excellence[21] reflect the complexity of these causal factors, and highlight the importance of adopting a multi-disciplinary approach to the assessment and management of violence within inpatient psychiatric settings. To date, the unique contribution of occupational therapists in this respect has not been systematically explored. Nevertheless, the wider literature concerning occupational therapy and violence risk assessment suggests three main ways in which occupational therapists are vital to reducing and managing violence.

Firstly, tools that are used to assess the risk of violence and/or recidivism incorporate risk and protective factors that are key areas of interest for occupational therapists. For example, the HCR-20[1] includes problems with employment and future living situation as risk factors for violence. These domains relate to core areas of interest for occupational therapists, namely an individual's capacity for work/education and their ability to live independently. Similarly, the Structured Assessment of Protective Factors (SAPROF)[22] includes other core occupational therapy concerns, for instance leisure activities, regular paid or unpaid work, and financial management. Such tools highlight that occupational therapists' efforts to develop individuals' skills for independent living, for meaningful education/employment and for satisfying leisure pursuits contribute directly to minimising violence risk factors and maximising protective factors. Although these tools may be used more commonly in forensic mental health settings, they do highlight the relationship between meaningful occupations and violence, irrespective of the civil or forensic background of patients. Indeed, the role of occupational therapists in assessing contextual factors for violence risk has been recognised by Blank[23], who argued that due to their expertise in this area, occupational therapists should be core contributors to violence risk assessment tools.

Secondly, occupational therapy theory identifies the importance of occupations for shaping a person's sense of identity. This is perhaps most apparent in Kielhofner's[24] idea of 'occupational identity', and Wilcock's mantra of 'doing, being and becoming'[25]. More recently, Twinley[26] has coined the phrase 'dark occupations' and explored the ways in which individuals engage in activities that, although harmful to oneself or others, can provide a sense of meaning, purpose, or identity. Indeed, she has argued that violence itself can be a meaningful occupation for individuals[27]. In contrast, Aldrich and White[28] have contested the idea of violence as a meaningful occupation, and rather frame it as an instrument of occupations and occupational identities (for example being a career criminal). Irrespective of whether violence is an occupation in itself, or an instrument associated with other occupations, understanding how it relates to a person's occupational identity cements the role of occupational therapists in providing alternative, pro-social patterns of occupation[27] that form the basis for new, violence-free identities.

Finally, occupational therapists contribute to reducing and managing violence through promoting successful occupational performance, which may be defined as the 'choosing, organizing and carrying out… [of] occupations in interaction with the environment'[29]. Occupational performance involves a

For peer review only - http://bmjopen.bmj.com/site/about/guidelines.xhtml

transactive and dynamic interplay between a person's abilities, their environment, and the demands of their chosen occupation[30]. Ultimately, this interplay either facilitates or inhibits an individual from completing activities and occupations successfully[30].

This transactive understanding can be applied to formulate violence within psychiatric inpatient settings. For example, an individual may be unable to progress to unescorted community leave and require an escort (the environmental factor) due to lacking emotional control (the intrapersonal factor) and therefore being verbally aggressive towards another person who is delaying them in a shop, instead of showing patience and consideration for others (the demands of the occupation). Thus, violence can be a direct consequence of a poor fit between a person's abilities, their environment, and the demands of their chosen occupation. Precedent exists for applying this transactive approach to violence risk assessment. Indeed, Rai[31] and Robbins[32] have demonstrated the relationship between impaired occupational performance and violence in the workplace and schools respectively. Both highlight the environmental and interpersonal factors that occupational therapists must treat in order to reduce violence, with Robbins[32] explicitly mentioning the 'goodness of fit' that is necessary between these factors. This understanding aligns with the biopsychosocial approach to violence that is prominent in current thinking. Nevertheless, it goes one step further by adding occupation to the equation, thus cementing the role of occupational therapists in preventing and managing violence.

In summary, the wider literature suggests an *a priori* model which explains how occupational therapists play a vital role in reducing and managing violence amongst mental health inpatients. Specifically, this model includes occupational therapists' role in: 1) decreasing the presence of risk factors and increasing the presence of protective factors, 2) re-shaping patterns of anti-social occupation and the identities these create and 3) reducing occupational dysfunction that results in violence.

STAGE ONE: IDENTIFYING THE RESEARCH QUESTION

Study rationale

Despite this tentative model, the actual contribution of occupational therapy to managing and reducing violence within mental health settings is currently ill-defined. Clarifying this contribution is essential if occupational therapists are to include violence reduction as a specific part of their efforts to improve patients' performance of – and participation in – essential and meaningful occupations, and thus promote their recovery from mental illness. Additionally, such efforts will have benefits beyond improving the health and well-being of those who commit violence. By reducing violence, occupational therapists may indirectly improve the health outcomes of its victims, reduce the use of restrictive practices, and lessen the financial costs of violence for mental health services.

Understanding the role of occupational therapy in this respect requires an overview of relevant, existing literature. This is best achieved through a scoping review, since such a review both maps existing knowledge and identifies gaps in this knowledge to inform future research priorities[33]. This review protocol is based on guidance from the Joanna Briggs Institute[34]. It also incorporates the six stages of a scoping review (as developed by Arksey and O'Malley[33] and subsequently enhanced by Levac, Colquhoun and O'Brien[35]) and guidance from the PRISMA Extension for Scoping Reviews checklist[36].

To prevent unnecessary duplication, a preliminary search for existing scoping and systematic reviews on the subject of occupational therapy and violence amongst mental health inpatients was carried out on 12th September 2020. The terms 'occupational therapy' AND 'violence', and 'occupational therapy'

For peer review only - http://bmjopen.bmj.com/site/about/guidelines.xhtml

AND ‘violence’ AND ‘mental health’ were used to search the JBI Evidence Synthesis, the Cochrane Database of Systematic Reviews, OTSeeker, PROSPERO, Google Scholar, CINAHL Plus, PsycINFO and AMED. No relevant reviews were identified.

Study aim and objectives

The aim of the review is to understand the role of occupational therapy in reducing and managing violence amongst mental health inpatients by addressing the following objectives:

1) to investigate how occupational therapy assessment contributes to predicting the risk of violence amongst mental health inpatients,

2) to explore how occupational therapy interventions can be used to decrease violence risk in inpatient mental health settings,

3) to examine the efficacy of occupational therapy assessment and intervention in reducing and managing violence amongst mental health inpatients.

STAGE TWO: IDENTIFYING RELEVANT STUDIES

Inclusion criteria

a) Population

As highlighted earlier in this paper, violence is a common occurrence in all mental health inpatient settings. Therefore, this review will include papers that focus on mental health inpatients irrespective of age, diagnosis, gender, civil or forensic status, and legal or voluntary status. Papers focussing on mental health outpatients will also be included. This is justified by the fact that relevant literature is anticipated to be limited and additional studies conducted within community settings may provide further information that addresses the study aim and objectives.

b) Concept

Studies that apply any aspect of an occupational therapist’s unique clinical reasoning to the assessment, formulation, reduction or management of violence committed by mental health inpatients will be included in this review. Essentially, relevant studies will seek to understand and/or treat violence from either an occupational therapy, or an occupational science, perspective. Studies which focus on discrete elements of function (for example emotional regulation) in relation to violence, but do not do so from an occupational perspective will be excluded. Additionally, self-injurious behaviours will be excluded since they fall outside the HCR-20 definition of violence[1].

c) Context

Since this study aims to map all available knowledge about the role of occupational therapy in reducing and managing violence amongst mental health inpatients, no exclusion criteria will be applied based on studies’ context. Indeed, the authors will include studies irrespective of context-specific factors, for example country of origin, system of healthcare or specific mental health inpatient setting.

d) Types of studies

Studies will not be excluded based on year of publication, type of research, research design or quality. Research from every level of the evidence-based hierarchy[37] will be included, provided it meets the inclusion criteria for study population and concept. Relevant studies include those that are peer-reviewed and published within formal academic journals, as well as grey literature, including, but not

For peer review only - http://bmjopen.bmj.com/site/about/guidelines.xhtml

limited to, conference presentations, policy documents and personal communications. Only studies written or available in English will be included.

Search strategy

In line with JBI guidance[34], this review will adopt a three-step search strategy. Firstly, the authors will search CINAHL Plus and PsycINFO for relevant studies, see Appendix 1 for the initial search strategy in CINAHL Plus. Since the reduction and management of violence relies on the expertise of various mental health professionals, relevant articles may be published in journals with a multi-disciplinary readership, or in journals aimed specifically at occupational therapists. These two databases index articles from a wide range of disciplines related to mental health, and have been chosen for their breadth and scope.

Both authors will analyse the keywords in the titles and abstracts of all retrieved papers, as well as the index terms assigned to each study. They will incorporate these terms into the original list of search terms to produce a final search strategy. In the second stage, this comprehensive strategy will be tailored to each of the following databases: CINAHL Plus, PsycINFO, MEDLINE, PsycARTICLES, ProQuest Health and Medicine, and AMED. Supplementary searching will be conducted in Google Scholar.

Following this search of the literature, the reference lists of all studies that will be included in the final review will be searched to identify additional relevant papers. This stage will also involve citation tracking and searches of grey literature. Grey literature searching will follow a three-stage process. Firstly, searches for relevant theses will be conducted in the following resources: ProQuest Dissertations and Theses Global, DART-Europe E-theses Portal, Global ETD Search, Microsoft Academic Search, Bielefeld Academic Search Engine and EThOS e-theses online service. These resources have been selected for the breadth of literature that they index collectively. Secondly, conference proceedings will be located. This will involve searching www.opengrey.eu and the webpages of key conferences focussing on violence and mental health. Additionally, wherever possible conference proceedings will be located in the databases used for the main literature search strategy. Finally, the authors of relevant papers (identified either through searches of academic or grey literature) will be contacted to identify further resources.

STAGE THREE: STUDY SELECTION

Selection process

The results of the literature search will be exported and collated in the Rayyan web app[38], and both authors will determine eligibility for each study. Initially, they will assess each result against the inclusion criteria using title/abstract alone. They will both do so independently and resolve any disagreements through consensus. Secondly, both authors will review the full-text of all articles selected for inclusion and determine eligibility against the inclusion criteria. Again, this will be done independently, and any disagreements will be resolved via consensus. The decision-making process of both authors will be made transparent through the inclusion in the final review of a PRISMA-ScR diagram[36], and a narrative description of the literature search and study selection processes. Both methods will detail reasons for the exclusion of full-text articles.

To promote consensus, the authors will pilot study selection using the process suggested by the JBI[34]. Specifically, twenty-five random abstracts will be screened independently by each author using the review's inclusion criteria. The authors will then discuss the decisions made during this screening and amend the inclusion criteria as necessary. Screening studies for inclusion will only start when both authors have reached a consensus in 75% of cases or more[34].

For peer review only - http://bmjopen.bmj.com/site/about/guidelines.xhtml

Critical Appraisal

Including a quality assessment in the proposed review risks divorcing the future research agenda from the realities of clinical practice and the lived experience of psychiatric inpatients and their families. Indeed, an evidence-based hierarchy will minimise 'poorer' quality evidence (for example opinion pieces or editorials) and thereby dismiss the concerns of key stakeholders that they contain. Ultimately such an approach may prevent services from being shaped by those who need them, and by those who are responsible for delivering them.

Such concerns may account for various guidelines omitting critical appraisal as a requirement for a scoping review (see, for example, Khalil et *al.* [39]). Nevertheless, critical appraisal can be considered a mark of quality[40] and necessary to ensure that scoping reviews provide meaningful evidence for policymaking, practice and research[41]. This review will appraise the quality of included papers using a simple checklist designed by Verhage and Boels[42] for use with quantitative, qualitative and mixed methods studies. 'Poorer' quality evidence will not be excluded, however the extent to which it influences and supports the review's findings will be identified using a sensitivity analysis[42].

STAGE FOUR: CHARTING THE DATA

A specific data-charting tool has been developed for the review, based on the tool provided in the JBI guidance for scoping reviews[34]. Charted data will include the following where relevant: author, location of study, date of publication, type of study, aims/purpose, sample size, gender of participants, participants' diagnoses, type of mental health inpatient setting (for example medium secure forensic unit, or psychiatric intensive care unit), which aspect of occupational therapy or occupational science theory is included, which element of treating violence (i.e. assessment / formulation / intervention / evaluation) is included, which aspect of the *a priori* model (explained in the introduction to this protocol) each study relates to, and key findings relevant to the review's aim and objectives.

To promote rigour, both authors will independently chart data for each study included in the review. The results of data charting will be discussed, and any disagreements resolved through consensus. As with the process of study selection, an initial pilot of data charting will be carried out using five studies. This will minimise any changes to the data-charting tool at a later stage.

STAGE FIVE: SUMMARISING AND REPORTING RESULTS

Findings will be analysed and presented using both qualitative and quantitative methods. Specifically, quantitative data will be analysed using frequency counts according to the categories presented above, and presented in tabular format, with key data also being presented in bar and/or pie charts. Qualitative data will be analysed using Carroll, Booth and Cooper's[43] 'best-fit' framework synthesis. Data will be coded to the pre-existing themes identified within the chosen *a priori* framework drawn from the wider risk management and occupational therapy literature. Additional data that does not map onto these existing themes will be analysed using secondary thematic analysis[44].

Both authors will perform this analysis independently, before reaching a consensus on which (if any) of the *a priori* themes, and which of the additional themes arising from secondary analysis are supported by the data. If sufficient data is available, all these themes will be used to construct a revised model explaining the role of occupational therapy in reducing and managing violence amongst mental health inpatients.

STAGE SIX: CONSULTATION

For peer review only - http://bmjopen.bmj.com/site/about/guidelines.xhtml

This scoping review forms the foundation for further research in this area. Its findings will undergo a period of consultation to ensure that the future research agenda is shaped by the perspectives and priorities of mental health inpatients, their families and the professionals involved in their care. Specifically, the consultation process will achieve this by validating and refining the review findings, and by giving them greater meaning through placing them into a more comprehensive perspective[35].

This consultation will involve a series of focus groups. Mental health inpatients and their families will participate in one set of focus groups, and mental health professionals from a variety of individual disciplines in another. These groups will be run in two stages. Initially, participants will be asked to reflect on their experiences and thoughts about the role of occupational therapy in reducing and managing violence and will do so before they are made aware of the review's findings (including the *a priori* and additional themes). Doing so will allow their views to be explored without risk of them being constrained or focussed on a particular direction. The findings of the review will then be shared with all participants and they will be asked to consider and discuss them.

All focus groups will be recorded and transcribed verbatim, and the resulting data will be analysed using thematic analysis[44]. Findings will then be examined in light of the review's original findings, and will either be incorporated into existing themes when they are congruent, or presented as additional themes and incorporated into the model. To promote transparency and rigour, the final review will account for any differences between the original analysis and the analysis after the consultation process.

ETHICS AND DISSEMINATION

The authors will disseminate the findings of this review during the consultation phase when the review is discussed with relevant stakeholders, and by submitting it for publication in a peer-reviewed academic journal. Since the review forms the foundation for additional research, additional dissemination may occur through the review being referenced in future academic papers.

With the exception of the consultation stage, the review will deal exclusively with secondary data and therefore will not require ethical approval. Such approval will be sought for the stakeholder consultation. Despite participants in this process acting as advisors rather than research participants[45], their insights will generate new data and ethical approval will ensure that this data is generated, processed and presented to robust standards.

For peer review only

For peer review only - http://bmjopen.bmj.com/site/about/guidelines.xhtml

REFERENCES

1 Douglas K S, Hart S D, Webster C D, et al. HCR-20 v3: Assessing risk for violence. Burnaby: Simon Fraser University 2013.

2 Thibaut B, Dewa L H, Ramtale S C, et al. Patient safety in inpatient mental health settings: a systematic review. *BMJ Open* 2019;9:1-19.

3 Dack C, Ross J, Papadopoulos C, et al. A review and meta-analysis of the patient factors associated with psychiatric in-patient aggression. *Acta Psychiatr Scand* 2013;127:255-268.

4 Flannery Jr R B, Wyshak G, and Flannery G J. Characteristics of international assaultive psychiatric patients: review of published findings, 2013-2017. *Psychiatric Quarterly* 2018;89:349-357.

5 Iozzino L, Ferrari C, Large M, et al. Prevalence and risk factors of violence by psychiatric acute inpatients: a systematic review and meta-analysis. *PLoS One* 2015;10:255-268.

6 Broderick C, Azizian, A, Kornbluh R, et al. Prevalence of physical violence in a forensic psychiatric hospital system during 2011-2013: patient assaults, staff assaults, and repeatedly violent patients. *CNS Spectr* 2015;20:319-330.

7 Flannery Jr R B, Wyshak G, Teece J, et al. Characteristics of International Assaultive Psychiatric Patients: Review of Published Findings, 2000-2012. *Psychiatric Quarterly* 2014;85:303-317.

8 Huitema A, Verstegen N, and de Vogel V. A study into the severity of forensic and civil inpatient aggression. *J Interpers Violence* 2018:1-19.

9 Lanctôt N, and Guay S. The aftermath of workplace violence among healthcare workers: a systematic literature review of the consequences. *Aggress Violent Behav* 2014;19:492-501.

10 Rubio-Valera M, Luciano J V, Ortiz J, et al. Health service use and costs associated with aggressiveness or agitation and containment in adult psychiatric care: a systematic review of the evidence. *BMC Psychiatry* 2015;15:35-47.

11 Chieze M, Hurst S, Kaiser S, et al. Effects of seclusion and restraint in adult psychiatry: a systematic review. *Front Psychiatry* 2019;10:491-509.

12 Serrano-Blanco A, Rubio-Valera M, Aznar-Lou I, et al. In-patient costs of agitation and containment in a mental health catchment area. *BMC Psychiatry* 2017;17:212-222.

13 de Loof P, Nijman H, Didden R, et al. Burnout symptoms in forensic psychiatric nurses and their associations with personality, emotional intelligence and client aggression: a cross-sectional study. *J Psychiatr Ment Health Nurs* 2018;25:506-516.

14 Pekurinen V, Välimäki M, Virtanen M, et al. Work stress and satisfaction with leadership among nurses encountering patient aggression in psychiatric care: a cross-sectional survey study. *Adm Policy Ment Health* 2019;46:368-379.

15 Cutcliffe J R, and Riahi, S. Systemic perspective of violence and aggression in mental health care: towards a more comprehensive understanding and conceptualization: part 2. *Int J Ment Health Nurs* 2013;22:568-578.

For peer review only - http://bmjopen.bmj.com/site/about/guidelines.xhtml

16 Cutcliffe J R, and Riahi, S. Systemic perspective of violence and aggression in mental health care: towards a more comprehensive understanding and conceptualization: part 1. *Int J Ment Health Nurs* 2013;22:558-567.

17 van der Schaff P S, Dusseldorp E, Keuning F M, et al. Impact of the physical environment of psychiatric wards on the use of seclusion. *B J Psych* 2013;202:142-149.

18 Jalil R, Huber J W, Sixsmith J, et al. The role of interpersonal style in aggression and its containment in a forensic mental health setting: a correlational and pseudoprospective study of patients and nursing staff. *Int J Ment Health Nurs* 2020;29:427-439.

19 Puzzo I, Aldridge-Waddon L, Bush E, et al. The relationship between ward social climate, ward sense of community, and incidents of disruptive behaviour: a study of a high secure psychiatric sample. *Int J Forensic Ment Health* 2019;18:153-163.

20 Nijman H L I. A model of aggression in psychiatric hospitals. *Acta Psychiatr Scand* 2002;106:142-143.

21 National Institute for Health and Care Excellence. Violence and aggression: short-term management in mental health, health and community settings. 2015. Available at: https://www.nice.org.uk/guidance/ng10 (Accessed 31 August 2020)

22 de Vogel V, de Ruiter C, Bouman Y, et al. SAPROF: guidelines for the assessment of protective factors for violence risk. English version. Utrecht: Forum Educatief 2007.

23 Blank A. Patient violence in community mental health: a review of literature. *Br J Occup Ther* 2001;64:584-859.

24 Kielhofner G. Model of human occupation: theory and application. 4th edn. Baltimore: Lippincott, Williams and Wilkins 2008.

25 Wilcock A. Reflections on doing, being and becoming. *Aust Occup Ther J* 1999;46:1-11.

26 Twinley R. The dark side of occupation: a concept for consideration. *Aust Occup Ther J* 2012;60:301-303.

27 Twinley R, and Addidle G. Considering violence: the dark side of occupation. *Br J Occup Ther* 2012;75:202-204.

28 Aldrich R M, and White N. Reconsidering violence: a response to Twinley and Addidle (2012) and Morris (2012). *Br J Occup Ther* 2012;75:527-529.

29 Creek J. The core concepts of occupational therapy practice: a dynamic framework for practice. London: Jessica Kingsley Publishers 2010.

30 Law M, Cooper B, Strong S, et al. The person-environment-occupation model: a transactive approach to occupational performance. *Can J Occup Ther* 1996;63:9-23.

31 Rai S. Preventing workplace aggression and violence: a role for occupational therapy. *Work* 2002;18:15-22.

32 Robbins J E. Violence prevention in schools: implications for occupational therapy. *Work* 2001;17:75-82.

For peer review only - http://bmjopen.bmj.com/site/about/guidelines.xhtml

33 Arksey H, and O'Malley L. Scoping studies: towards a methodological framework. *Int J Soc Res Methodol* 2005;8:19-32.

34 Peters M D J, Godfrey C, McInerney P, et al. Chapter 11: Scoping Reviews. In: Aromataris E, and Munn Z, eds. Joanna Briggs Institute Reviewer's Manual. 2020. Available at: https://reviewersmanual.joannabriggs.org (Accessed 13 June 2020).

35 Levac D, Colquhoun H, and O'Brien K K. Scoping studies: advancing the methodology. *Implementation Science* 2010;5:69-77.

36 Tricco A C, Lillie E, Zarin W, et al. PRISMA Extension for scoping reviews (PRISMA-ScR) checklist and explanation. *Ann Intern Med* 2018;169:467-473.

37 Tomlin G, and Borgetto B. Research pyramid: a new evidence-based practice model for occupational therapy. *Am J Occup Ther* 2011;65:189-196.

38 Ouzzani M, Hammady H, Fedorowicz Z, et al. Rayyan: a web and mobile app for systematic reviews. *Syst Rev* 2016;5:210-219.

39 Khalil H, Peters M, Godfrey C M, et al. An evidence-based approach for scoping reviews. *Worldviews Evid Based Nurs* 2016;13:118-123.

40 Cooper S, Cant R, Kelly M, et al. An evidence-based checklist for improving scoping review quality. *Clin Nurs Res* 2019:1-24.

41 Daudt H M, van Mossel C, and Scott S J. Enhancing the scoping study methodology: a large, inter-professional team's experience with Arksey and O'Malley's framework. *BMC Med Res Methodol* 2013;13:48-56.

42 Verhage A, and Boels, D. Critical appraisal of mixed methods research studies in a systematic scoping review on plural policing: assessing the impact of excluding inadequately reported studies by means of a sensitivity analysis. *Qual Quant* 2017:51;1449-1468.

43 Carroll C, Booth A, and Cooper K. A worked example of 'best-fit' framework synthesis: a systematic review of views concerning the taking of some potential chemopreventative agents. *BMC Med Res Methodol* 2011;11:21-29.

44 Braun B, and Clarke V. Using thematic analysis in psychology. *Qual Res Psychol* 2006;3:77-101.

45 National Institute for Health Research. Do I need to apply for ethical approval to involve the public in my research?'. No date. Available at: https://www.invo.org.uk/posttypefaq/do-i-need-to-apply-for-ethical-approval-to-involve-the-public-in-my-research (Accessed 13 June 2020)

Author contributions: Both DB and NH were involved in designing the protocol. DB prepared the manuscript for publication.

Funding statement: This work was completed as part of The Health Education England and National Institute for Health Research Integrated Clinical Academic Programme.

Competing interests statement: none declared.

For peer review only - http://bmjopen.bmj.com/site/about/guidelines.xhtml

Appendix One – Literature Search Strategy for CINAHL Plus

Search	Date	Query	Limiters	Result
S4	08/09/20	S1 and S2 and S3	Subject field<br><br>Studies in English	10,528
S3	08/09/20	mental illness OR mental disorder* OR mental*health in*patient OR schizophrenia OR schizophrenic OR schizoaffective disorder OR bi-polar disorder OR manic depression OR depression OR psychosis OR psychotic OR neurosis OR neuroses OR neurotic OR general anxiety disorder OR anxiety disorder OR personality disorder OR paranoid personality disorder OR schizoid personality disorder OR schizotypal personality disorder OR antisocial personality disorder OR borderline personality disorder OR histrionic personality disorder OR narcissistic personality disorder OR avoidant personality disorder OR dependent personality disorder OR obsessive-compulsive personality disorder OR dissocial personality disorder OR emotionally unstable personality disorder OR anankastic personality disorder OR anxious personality disorder OR psychopath* OR learning disabilit* OR learning difficult* OR learning impair* OR mental retardation OR mentally retarded OR intellectual disability OR intellectual difficulty OR intellectual retardation OR Aspergers OR Aspergers Syndrome OR autism OR autistic disorder OR developmental disability OR traumatic brain injury OR TBI OR acquired brain injury OR ABI OR neuropsychiatr* OR dementia OR demented OR Alzheimer's OR Alzheimer's disease OR alcoholic* OR alcohol abuse OR alcohol misuse OR drug addict OR drug user OR drug abuse OR drug misuse OR substance abuse OR substance misuse OR paraphilia* OR mentally disordered OR mentally*disordered offender OR mentally ill offender OR psychiatric intensive care unit OR PICU or acute mental health OR mental health rehabilitation OR forensic mental health OR chronic mental health OR healthcare wing OR psychiatric setting OR axis 1 diagnosis OR acute inpatient psychiatric unit OR locked	Subject field<br><br>Studies in English	377,962

For peer review only

		rehabilitation unit OR locked rehabilitation ward OR secure unit OR secure hospital		
S2	08/09/20	forensic risk assessment OR forensic risk reduction OR forensic risk formulation OR violence risk OR violence formulation OR seclusion OR secluded OR long*term segregation OR extra*care OR enhanced observations OR de*seclusion OR de*secluded OR physical restraint OR mechanical restraint OR chemical restraint OR rapid tranquilisation OR aggression OR violence OR agitation OR hostility OR violence risk OR aggressive behavio*rs OR violent behavio*rs OR risk behavio*rs OR violent ideation OR aggression prevention OR violence prevention OR violence control OR emotional regulation OR emotional dysregulation OR acute arousal OR mental health crisis OR assault OR HCR-20 OR Historical Clinical Risk Management*20 OR RSVP OR Risk for Sexual Violence Protocol OR SAPROF OR Structured Assessment of Protective Factors for Risk of Violence OR SAVRY OR Structured Assessment of Violence Risk in Youth OR START OR Short Term Assessment of Risk and Treatability OR HoNOS OR Health of the Nation Outcome Scale OR HoNOS*secure OR Health of the Nation Outcome Scale for Users of Secure and Forensic Services OR offending behaviour OR offending OR re*offending OR offence paralleling behaviour OR recidivism OR staff observed aggression scale OR SOAS OR Brøset Violence Checklist	Subject field<br><br>Studies in English	3,597,064
S1	08/09/20	occupational therapy OR occupational therapist OR occupational science OR occupational scientist OR occupational focus OR occupationally focus* OR occupational perspective OR occupational therapy assessment OR occupational therapy risk assessment OR occupational performance OR occupational participation OR functional ability OR functional performance OR occupational identity OR occupational role OR occupational alienation OR occupational deprivation OR occupational disruption OR occupational dysfunction OR occupational imbalance OR occupational adaptation OR occupational justice OR occupational injustice OR dark therap* OR dark occupation OR living skills OR self*care OR personal care OR activities of daily living OR ADLs OR instrumental activities of daily living OR IADLs	Subject field<br><br>Studies in English	76,630

For peer review only

		OR personal activities of daily living OR PADLs OR basic activities of daily living OR BADLs OR vocational rehabilitation OR sensory assessment OR sensory approach OR sensory*informed approach OR sensory integration OR sensory integrative OR sensory intervention OR sensory modulation OR sensory processing OR sensory stimulation OR sensory profile OR sensory defensiveness OR sensory overload OR sensory overstimulation OR sensory room OR Model of Human Occupation OR MOHO OR Canadian Model of Occupational Performance and Engagement OR CMOP-E OR Person*Environment*Occupation*Performance model OR PEOP OR Vona du Toit Model of Creative Ability OR VdTMoCA OR Kawa OR Kawa model		

For peer review only

BMJ Open

The Role of Occupational Therapy in Reducing and Managing Violence amongst Mental Health Inpatients: A Scoping Review Protocol

Journal:	*BMJ Open*
Manuscript ID	bmjopen-2020-046260.R1
Article Type:	Protocol
Date Submitted by the Author:	07-Jun-2021
Complete List of Authors:	Bell, David; University of Birmingham; St Andrew's Healthcare, Occupational Therapy Department<br>Hallett, Nutmeg; University of Birmingham , School of Nursing
**Primary Subject Heading**:	Mental health
Secondary Subject Heading:	Mental health
Keywords:	PSYCHIATRY, MENTAL HEALTH, FORENSIC MEDICINE

SCHOLARONE™
Manuscripts

I, the Submitting Author has the right to grant and does grant on behalf of all authors of the Work (as defined in the below author licence), an exclusive licence and/or a non-exclusive licence for contributions from authors who are: i) UK Crown employees; ii) where BMJ has agreed a CC-BY licence shall apply, and/or iii) in accordance with the terms applicable for US Federal Government officers or employees acting as part of their official duties; on a worldwide, perpetual, irrevocable, royalty-free basis to BMJ Publishing Group Ltd ("BMJ") its licensees and where the relevant Journal is co-owned by BMJ to the co-owners of the Journal, to publish the Work in this journal and any other BMJ products and to exploit all rights, as set out in our licence.

The Submitting Author accepts and understands that any supply made under these terms is made by BMJ to the Submitting Author unless you are acting as an employee on behalf of your employer or a postgraduate student of an affiliated institution which is paying any applicable article publishing charge ("APC") for Open Access articles. Where the Submitting Author wishes to make the Work available on an Open Access basis (and intends to pay the relevant APC), the terms of reuse of such Open Access shall be governed by a Creative Commons licence – details of these licences and which Creative Commons licence will apply to this Work are set out in our licence referred to above.

Other than as permitted in any relevant BMJ Author's Self Archiving Policies, I confirm this Work has not been accepted for publication elsewhere, is not being considered for publication elsewhere and does not duplicate material already published. I confirm all authors consent to publication of this Work and authorise the granting of this licence.

For peer review only

The Role of Occupational Therapy in Reducing and Managing Violence amongst Mental Health Inpatients: A Scoping Review Protocol

Corresponding author:

David Bell, Occupational Therapist, St Andrew's Healthcare, 70 Dogpool Lane, Birmingham. United Kingdom. B30 2XR dsbell@standrew.co.uk

AND

Health Education England and National Institute for Health Research Integrated Clinical Academic Intern

ORCID ID: https://orcid.org/0000-0002-8693-0537

Co-author:

Nutmeg Hallett, PhD, Lecturer in Mental Health Nursing, University of Birmingham, The School of Nursing, College of Medical and Dental Sciences, Edgbaston, Birmingham. United Kingdom. B15 2TT.

ORCID ID: https://orcid.org/0000-0003-3115-8831

Word count:

3408

For peer review only - http://bmjopen.bmj.com/site/about/guidelines.xhtml

ABSTRACT

Introduction: Violence is pervasive amongst psychiatric inpatients and has profound consequences for its victims, its perpetrators and mental health services. Currently, the unique contribution of occupational therapists to reducing and managing violence amongst this patient group has not been systematically explored. However, an *a priori* model which provides an initial understanding of its role in this respect can be identified from the wider scholarly literature. This scoping review aims to apply and refine this model, thereby producing an amended version that will form the basis for further research.

Methods and analysis: This scoping review is based upon guidance from the Joanna Briggs Institute, Levac, Colquhoun and O'Brien's scoping review framework, and the Preferred Reporting Items for Systematic Reviews and Meta-Analyses Scoping Review checklist. Electronic databases (CINAHL Plus, PsycINFO, MEDLINE, PsycARTICLES, ProQuest Health and Medicine, AMED and Google Scholar) and grey literature will be searched to identify relevant papers. Included articles will apply occupational therapy theory or occupational science to the reduction or management of violence among psychiatric inpatients and will be critically appraised by two independent reviewers. Study characteristics will be presented using frequency counts, and qualitative data will be analysed using 'best-fit' framework synthesis and secondary thematic analysis to produce an overall model of occupational therapy's contribution to violence management and reduction.

Ethics and dissemination: Results will be disseminated through a peer-reviewed academic journal and via professional conferences. The review will collect secondary data and therefore will not require ethical approval.

STRENGTHS AND LIMITATIONS OF THIS STUDY

- This study is the first attempt to map out the unique role of occupational therapy in reducing and managing violence in psychiatric inpatient settings.
- Using a combination of 'best-fit' framework synthesis and secondary thematic analysis will provide a model that organises and makes explicit existing knowledge in this respect.
- Excluding self-harm as an act of violence may result in relevant studies being omitted from the final review.

KEYWORDS

Occupational therapy, occupational science, violence, aggression, mental health inpatients, best-fit framework synthesis

For peer review only - http://bmjopen.bmj.com/site/about/guidelines.xhtml

INTRODUCTION

Violence may be defined as 'an act [committed] with some degree of wilfulness that caused/has the potential to cause physical or serious psychological harm to another person/persons'[1]. Within mental health inpatient settings, violent acts are both chronic and pervasive. Indeed, a recent systematic review of patient safety within these settings reported that 43% of participants (staff and patients) had suffered physical violence and 57% verbal aggression[2].

Whilst evidence from systematic reviews suggests that psychiatric inpatients who commit acts of aggression and violence are most likely to be younger, male, and involuntarily admitted with a diagnosis of schizophrenia[3-4], other studies report high rates of violence amongst entire psychiatric populations. For example, Iozzino et *al's.*[5] meta-analysis reported that nearly 20% of psychiatric acute inpatients display acts of physical violence during their treatment in hospital. Similarly, Broderick et *al*.[6] reported that 31% of patients in a large, multihospital forensic system committed at least one physically violent assault during their hospitalisation. Furthermore, additional studies have shown that violence is a common feature of psychiatric inpatient settings, irrespective of country[2, 5, 7], patients' gender[7-8], patients' diagnosis[7-8] and civil or forensic status[2, 8]. Thus, whilst psychiatric inpatients who act aggressively and violently may commonly fit a particular demographic, violence is by no means the preserve of a small group of psychiatric inpatients.

Given its prevalence, violence in psychiatric inpatient settings inevitably has severe and far-reaching consequences. This is most obviously the case for its victims, who have reported increased physical health problems, decreased emotional well-being and various psychological impairments, most commonly symptoms of post-traumatic stress disorder[9]. Whilst healthcare professionals and patients can both be affected directly in this manner, patients experience further suffering through the reduction in the quality of care they receive from assaulted healthcare workers[9].

Additionally, the perpetrators of violence experience negative outcomes. A recent systematic review concluded that there is some evidence to suggest that agitated and aggressive psychiatric inpatients experience longer periods of admission, increased probability of readmission and increased medication usage[10]. Moreover, they are likely to be affected by restrictive practices, for example seclusion, or chemical or physical restraint, which are used to contain violence and aggression. These interventions are associated with post-traumatic stress disorder in 25%-47% of cases, as well as predominantly negative emotions, including distress and feeling punished[11].

Finally, violence has negative consequences for healthcare services. Practices to contain violence entail significant financial costs, calculated at 822€ per episode[12]. Additionally, patient aggression has been correlated with burnout in forensic psychiatric nurses[13], higher levels of work stress amongst forensic nurses[14], and decreased job satisfaction among healthcare workers[9]. Such factors inevitably increase the financial burden on services, through reduced effectiveness and retention of staff.

The prevalence and impact of violence amongst psychiatric inpatients make it unsurprising that considerable efforts have been made to understand its causal factors. Historically, the dominance of the medical approach within healthcare services has meant that violence has been understood as having pathological origins[15], with its roots in patients' mental illness and personality structures. In contrast, more recent understandings have emphasised the importance of environmental factors and more specifically, their complex interactions with these patient-related factors[15-16]. Thus, recent efforts to understand violence in psychiatric settings have been far more diverse in their scope, and have sought to identify how violence is influenced by (amongst other factors), the physical

For peer review only - http://bmjopen.bmj.com/site/about/guidelines.xhtml

environments of wards[17], nurse and patient interpersonal styles[18], ward social climate and sense of community[19], and coercive measures[15]. Perhaps most comprehensive in this respect is Cutcliffe's and Riahi's[15-16] systemic model of variables that contribute to aggression and violence, which incorporates phenomena related to the environment, individual patients, individual clinicians and a mental health-care system in its entirety. Similarly, Nijman[20] presents a model that identifies patient, ward and environmental factors that can cause violence, however this model outlines more explicitly the interplay between these factors, and the 'vicious circle' whereby they interact not only to cause violence, but to reinforce it. Both of these models highlight the shift away from using a medical approach to understand violence, towards using a biopsychosocial approach.

Guidelines from the National Institute for Health and Care Excellence[21] reflect the complexity of these causal factors, and highlight the importance of adopting a multi-disciplinary approach to the assessment and management of violence within inpatient psychiatric settings. To date, the unique contribution of occupational therapists in this respect has not been systematically explored. This is surprising, since they are core members of multidisciplinary teams within these settings, and use rich and diverse theoretical frameworks[22] to promote individuals' ability to perform meaningful occupations. These patterns of meaningful occupations are a central part of recovery from mental illness, due to their importance for providing an individual with a sense of purpose and a clearly defined personal identity, both of which enable participation in society[23]. Since this process of recovery from mental illness is undermined by violence, its reduction must be a key concern for occupational therapists. Despite the lack of evidence regarding their contribution in this respect, the wider literature concerning occupational therapy and violence risk assessment suggests three main ways in which occupational therapists are vital to reducing and managing violence.

Firstly, tools that are used to assess the risk of violence and/or recidivism incorporate risk and protective factors that are key areas of interest for occupational therapists. For example, the HCR-20[1] includes problems with employment and future living situation as risk factors for violence. These domains relate to core areas of interest for occupational therapists, namely an individual's capacity for work/education and their ability to live independently. Similarly, the Structured Assessment of Protective Factors (SAPROF)[24] includes other core occupational therapy concerns, for instance leisure activities, regular paid or unpaid work, and financial management. Such tools highlight that occupational therapists' efforts to develop individuals' skills for independent living, for meaningful education/employment and for satisfying leisure pursuits contribute directly to minimising violence risk factors and maximising protective factors. Although these tools may be used more commonly in forensic mental health settings, they do highlight the relationship between meaningful occupations and violence, irrespective of the civil or forensic background of patients. Indeed, the role of occupational therapists in assessing contextual factors for violence risk has been recognised by Blank[25], who argued that due to their expertise in this area, occupational therapists should be core contributors to violence risk assessment tools.

Secondly, occupational therapy theory identifies the importance of occupations for shaping a person's sense of identity. This is perhaps most apparent in Kielhofner's[26] idea of 'occupational identity', and Wilcock's mantra of 'doing, being and becoming'[27]. More recently, Twinley[28] has coined the phrase 'dark occupations' and explored the ways in which individuals engage in activities that, although harmful to oneself or others, can provide a sense of meaning, purpose, or identity. Indeed, she has argued that violence itself can be a meaningful occupation for individuals[29]. In contrast, Aldrich and White[30] have contested the idea of violence as a meaningful occupation, and rather frame it as an instrument of occupations and occupational identities (for example being a career criminal). Irrespective of whether violence is an occupation in itself, or an instrument associated with

For peer review only - http://bmjopen.bmj.com/site/about/guidelines.xhtml

For peer review only

other occupations, understanding how it relates to a person's occupational identity cements the role of occupational therapists in providing alternative, pro-social patterns of occupation[29] that form the basis for new, violence-free identities.

Finally, occupational therapists contribute to reducing and managing violence through promoting successful occupational performance, which may be defined as the 'choosing, organizing and carrying out… [of] occupations in interaction with the environment'[23]. Occupational performance involves a transactive and dynamic interplay between a person's abilities, their environment, and the demands of their chosen occupation[31]. Ultimately, this interplay either facilitates or inhibits an individual from completing activities and occupations successfully[31].

This transactive understanding can be applied to formulate violence within psychiatric inpatient settings. For example, an individual may be unable to progress to unescorted community leave and require an escort (the environmental factor) due to lacking emotional control (the intrapersonal factor) and therefore being verbally aggressive towards another person who is delaying them in a shop, instead of showing patience and consideration for others (the demands of the occupation). Thus, violence can be a direct consequence of a poor fit between a person's abilities, their environment, and the demands of their chosen occupation. Precedent exists for applying this transactive approach to violence risk assessment. Indeed, Rai[32] and Robbins[33] have demonstrated the relationship between impaired occupational performance and violence in the workplace and schools respectively. Both highlight the environmental and interpersonal factors that occupational therapists must treat in order to reduce violence, with Robbins[33] explicitly mentioning the 'goodness of fit' that is necessary between these factors. This understanding aligns with the biopsychosocial approach to violence that is prominent in current thinking. Nevertheless, it goes one step further by adding occupation to the equation, thus cementing the role of occupational therapists in preventing and managing violence.

In summary, the wider literature suggests an *a priori* model which explains how occupational therapists play a vital role in reducing and managing violence amongst mental health inpatients. Specifically, this model includes occupational therapists' role in: 1) decreasing the presence of risk factors and increasing the presence of protective factors, 2) re-shaping patterns of anti-social occupation and the identities these create and 3) reducing occupational dysfunction that results in violence.

STAGE ONE: IDENTIFYING THE RESEARCH QUESTION

Study rationale

Despite this tentative model, the actual contribution of occupational therapy to managing and reducing violence within mental health settings is currently ill-defined. Clarifying this contribution is essential if occupational therapists are to include violence reduction as a specific part of their efforts to improve patients' performance of – and participation in – essential and meaningful occupations, and thus promote their recovery from mental illness. Additionally, such efforts will have benefits beyond improving the health and well-being of those who commit violence. By reducing violence, occupational therapists may indirectly improve the health outcomes of its victims, reduce the use of restrictive practices, and lessen the financial costs of violence for mental health services.

Understanding the role of occupational therapy in this respect requires an overview of relevant, existing literature. This is best achieved through a scoping review, since such a review both maps existing knowledge and identifies gaps in this knowledge to inform future research priorities[34]. This review protocol is based on guidance from the Joanna Briggs Institute[35]. It also incorporates five of

For peer review only - http://bmjopen.bmj.com/site/about/guidelines.xhtml

the six stages of a scoping review (as developed by Arksey and O'Malley[34] and subsequently enhanced by Levac, Colquhoun and O'Brien[36]) and guidance from the PRISMA Extension for Scoping Reviews checklist[37].

To prevent unnecessary duplication, a preliminary search for existing scoping and systematic reviews on the subject of occupational therapy and violence amongst mental health inpatients was carried out on 12th September 2020. The terms 'occupational therapy' AND 'violence', and 'occupational therapy' AND 'violence' AND 'mental health' were used to search the JBI Evidence Synthesis, the Cochrane Database of Systematic Reviews, OTSeeker, PROSPERO, Google Scholar, CINAHL Plus, PsycINFO and AMED. No relevant reviews were identified.

Patient and Public Involvement

Unfortunately, it has not been possible to involve either the public or patients in the design of the proposed review.

Study aim and objectives

The aim of the review is to understand the role of occupational therapy in reducing and managing violence amongst mental health inpatients, and in so doing, to inform future research priorities. This aim will be achieved by addressing the following objectives:

1) to investigate how occupational therapy assessment contributes to predicting the risk of violence amongst mental health inpatients,

2) to explore how occupational therapy interventions can be used to decrease violence risk in inpatient mental health settings.

STAGE TWO: IDENTIFYING RELEVANT STUDIES

Inclusion criteria

a) Population

As highlighted earlier in this paper, violence is a common occurrence in all mental health inpatient settings. Therefore, this review will include papers that focus on mental health inpatients irrespective of age, diagnosis, gender, civil or forensic status, and legal or voluntary status. Papers focussing on mental health outpatients will also be included. This is justified by the fact that relevant literature is anticipated to be limited and additional studies conducted within community settings may provide further information that addresses the study aim and objectives.

b) Concept

Studies that apply any aspect of an occupational therapist's unique clinical reasoning to the assessment, formulation, reduction or management of violence committed by mental health inpatients will be included in this review. Essentially, relevant studies will seek to understand and/or treat violence from either an occupational therapy, or an occupational science, perspective. Studies which focus on discrete elements of function (for example emotional regulation) in relation to violence, but do not do so from an occupational perspective will be excluded. Additionally, self-injurious behaviours will be excluded since they fall outside the HCR-20 definition of violence[1].

c) Context

For peer review only - http://bmjopen.bmj.com/site/about/guidelines.xhtml

Since this study aims to map all available knowledge about the role of occupational therapy in reducing and managing violence amongst mental health inpatients, no exclusion criteria will be applied based on studies' context. Indeed, the authors will include studies irrespective of context-specific factors, for example country of origin, system of healthcare or specific mental health inpatient setting.

d) Types of studies

Studies will not be excluded based on year of publication, type of research, research design or quality. Research from every level of the evidence-based hierarchy[38] will be included, provided it meets the inclusion criteria for study population and concept. Relevant studies include those that are peer-reviewed and published within formal academic journals, as well as grey literature, including, but not limited to, conference presentations, policy documents and personal communications. Only studies written or available in English will be included.

Search strategy

In line with JBI guidance[35], this review will adopt a three-step search strategy. Firstly, the authors will search CINAHL Plus and PsycINFO for relevant studies, see Appendix 1 for the initial search strategy in CINAHL Plus. Since the reduction and management of violence relies on the expertise of various mental health professionals, relevant articles may be published in journals with a multi-disciplinary readership, or in journals aimed specifically at occupational therapists. These two databases index articles from a wide range of disciplines related to mental health, and have been chosen for their breadth and scope.

Both authors will analyse the keywords in the titles and abstracts of all retrieved papers, as well as the index terms assigned to each study. They will incorporate these terms into the original list of search terms to produce a final search strategy. In the second stage, this comprehensive strategy will be tailored to each of the following databases: CINAHL Plus, PsycINFO, MEDLINE, PsycARTICLES, ProQuest Health and Medicine, and AMED. Supplementary searching will be conducted in Google Scholar.

Following this search of the literature, the reference lists of all studies that will be included in the final review will be searched to identify additional relevant papers. This stage will also involve citation tracking and searches of grey literature. Grey literature searching will follow a three-stage process. Firstly, searches for relevant theses will be conducted in the following resources: ProQuest Dissertations and Theses Global, DART-Europe E-theses Portal, Global ETD Search, Microsoft Academic Search, Bielefeld Academic Search Engine and EThOS e-theses online service. These resources have been selected for the breadth of literature that they index collectively. Secondly, conference proceedings will be located. This will involve searching www.opengrey.eu and the webpages of key conferences focussing on violence and mental health. Additionally, wherever possible conference proceedings will be located in the databases used for the main literature search strategy. Finally, the authors of relevant papers (identified either through searches of academic or grey literature) will be contacted to identify further resources.

STAGE THREE: STUDY SELECTION

Selection process

The results of the literature search will be exported and collated in the Rayyan web app[39], and both authors will determine eligibility for each study. Initially, they will assess each result against the inclusion criteria using title/abstract alone. They will both do so independently and resolve any disagreements through consensus. Secondly, both authors will review the full-text of all articles selected for inclusion and determine eligibility against the inclusion criteria. Again, this will be done

For peer review only - http://bmjopen.bmj.com/site/about/guidelines.xhtml

independently, and any disagreements will be resolved via consensus. The decision-making process of both authors will be made transparent through the inclusion in the final review of a PRISMA-ScR diagram[37], and a narrative description of the literature search and study selection processes. Both methods will detail reasons for the exclusion of full-text articles.

To promote consensus, the authors will pilot study selection using the process suggested by the JBI[35]. Specifically, twenty-five random abstracts will be screened independently by each author using the review's inclusion criteria. The authors will then discuss the decisions made during this screening and amend the inclusion criteria as necessary. Screening studies for inclusion will only start when both authors have reached a consensus in 75% of cases or more[35].

Critical Appraisal

Including a quality assessment in the proposed review risks divorcing the future research agenda from the realities of clinical practice and the lived experience of psychiatric inpatients and their families. Indeed, an evidence-based hierarchy will minimise 'poorer' quality evidence (for example Eopinion pieces or editorials) and thereby dismiss the concerns of key stakeholders that they contain. Ultimately such an approach may prevent services from being shaped by those who need them, and by those who are responsible for delivering them.

Such concerns may account for various guidelines omitting critical appraisal as a requirement for a scoping review (see, for example, Khalil et *al.* [40]). Nevertheless, critical appraisal can be considered a mark of quality[41] and necessary to ensure that scoping reviews provide meaningful evidence for policymaking, practice and research[42]. This review will appraise the quality of included papers using a simple checklist designed by Verhage and Boels[43] for use with quantitative, qualitative and mixed methods studies. 'Poorer' quality evidence will not be excluded, however the extent to which it influences and supports the review's findings will be identified using a sensitivity analysis[43].

STAGE FOUR: CHARTING THE DATA

A specific data-charting tool has been developed for the review, based on the tool provided in the JBI guidance for scoping reviews[35]. Charted data will include the following where relevant: author, location of study, date of publication, type of study, aims/purpose, sample size, gender of participants, participants' diagnoses, type of mental health inpatient setting (for example medium secure forensic unit, or psychiatric intensive care unit), which aspect of occupational therapy or occupational science theory is included, which element of treating violence (i.e. assessment / formulation / intervention / evaluation) is included, which aspect of the *a priori* model (explained in the introduction to this protocol) each study relates to, and key findings relevant to the review's aim and objectives.

To promote rigour, both authors will independently chart data for each study included in the review. The results of data charting will be discussed, and any disagreements resolved through consensus. As with the process of study selection, an initial pilot of data charting will be carried out using five studies. This will minimise any changes to the data-charting tool at a later stage.

STAGE FIVE: SUMMARISING AND REPORTING RESULTS

Findings will be analysed and presented using both qualitative and quantitative methods. Specifically, quantitative data will be analysed using frequency counts according to the categories presented above, and presented in tabular format, with key data also being presented in bar and/or pie charts. Qualitative data will be analysed using Carroll, Booth and Cooper's[44] 'best-fit' framework synthesis. Data will be coded to the pre-existing themes identified within the chosen *a priori* framework drawn

For peer review only - http://bmjopen.bmj.com/site/about/guidelines.xhtml

from the wider risk management and occupational therapy literature. Additional data that does not map onto these existing themes will be analysed using secondary thematic analysis[45].

Both authors will perform this analysis independently, before reaching a consensus on which (if any) of the *a priori* themes, and which of the additional themes arising from secondary analysis are supported by the data. If sufficient data is available, all these themes will be used to construct a revised model explaining the role of occupational therapy in reducing and managing violence amongst mental health inpatients.

ETHICS AND DISSEMINATION

The authors will disseminate the findings of this review by submitting it for publication in a peer-reviewed academic journal and via professional conferences. Since the review forms the foundation for additional research, additional dissemination may occur through the review being referenced in future academic papers. The review itself will deal exclusively with secondary data and therefore will not require ethical approval.

Author contributions: Both DB and NH were involved in designing the protocol. DB prepared the manuscript for publication.

Funding statement: This work was completed as part of The Health Education England and National Institute for Health Research Integrated Clinical Academic Programme. Grant/award number: not applicable.

Competing interests statement: none declared.

For peer review only

For peer review only - http://bmjopen.bmj.com/site/about/guidelines.xhtml

REFERENCES

1 Douglas K S, Hart S D, Webster C D, et al. HCR-20 v3: Assessing risk for violence. Burnaby: Simon Fraser University 2013.

2 Thibaut B, Dewa L H, Ramtale S C, et al. Patient safety in inpatient mental health settings: a systematic review. *BMJ Open* 2019;9:1-19.

3 Dack C, Ross J, Papadopoulos C, et al. A review and meta-analysis of the patient factors associated with psychiatric inpatient aggression. *Acta Psychiatr Scand* 2013;127:255-268.

4 Flannery Jr R B, Wyshak G, and Flannery G J. Characteristics of international assaultive psychiatric patients: review of published findings, 2013-2017. *Psychiatric Quarterly* 2018;89:349-357.

5 Iozzino L, Ferrari C, Large M, et al. Prevalence and risk factors of violence by psychiatric acute inpatients: a systematic review and meta-analysis. *PLoS One* 2015;10:255-268.

6 Broderick C, Azizian, A, Kornbluh R, et al. Prevalence of physical violence in a forensic psychiatric hospital system during 2011-2013: patient assaults, staff assaults, and repeatedly violent patients. *CNS Spectr* 2015;20:319-330.

7 Flannery Jr R B, Wyshak G, Teece J, et al. Characteristics of International Assaultive Psychiatric Patients: Review of Published Findings, 2000-2012. *Psychiatric Quarterly* 2014;85:303-317.

8 Huitema A, Verstegen N, and de Vogel V. A study into the severity of forensic and civil inpatient aggression. *J Interpers Violence* 2018:1-19.

9 Lanctôt N, and Guay S. The aftermath of workplace violence among healthcare workers: a systematic literature review of the consequences. *Aggress Violent Behav* 2014;19:492-501.

10 Rubio-Valera M, Luciano J V, Ortiz J, et al. Health service use and costs associated with aggressiveness or agitation and containment in adult psychiatric care: a systematic review of the evidence. *BMC Psychiatry* 2015;15:35-47.

11 Chieze M, Hurst S, Kaiser S, et al. Effects of seclusion and restraint in adult psychiatry: a systematic review. *Front Psychiatry* 2019;10:491-509.

12 Serrano-Blanco A, Rubio-Valera M, Aznar-Lou I, et al. Inpatient costs of agitation and containment in a mental health catchment area. *BMC Psychiatry* 2017;17:212-222.

13 de Loof P, Nijman H, Didden R, et al. Burnout symptoms in forensic psychiatric nurses and their associations with personality, emotional intelligence and client aggression: a cross-sectional study. *J Psychiatr Ment Health Nurs* 2018;25:506-516.

14 Pekurinen V, Välimäki M, Virtanen M, et al. Work stress and satisfaction with leadership among nurses encountering patient aggression in psychiatric care: a cross-sectional survey study. *Adm Policy Ment Health* 2019;46:368-379.

15 Cutcliffe J R, and Riahi, S. Systemic perspective of violence and aggression in mental health care: towards a more comprehensive understanding and conceptualization: part 2. *Int J Ment Health Nurs* 2013;22:568-578.

For peer review only - http://bmjopen.bmj.com/site/about/guidelines.xhtml

16 Cutcliffe J R, and Riahi, S. Systemic perspective of violence and aggression in mental health care: towards a more comprehensive understanding and conceptualization: part 1. *Int J Ment Health Nurs* 2013;22:558-567.

17 van der Schaff P S, Dusseldorp E, Keuning F M, et al. Impact of the physical environment of psychiatric wards on the use of seclusion. *B J Psych* 2013;202:142-149.

18 Jalil R, Huber J W, Sixsmith J, et al. The role of interpersonal style in aggression and its containment in a forensic mental health setting: a correlational and pseudoprospective study of patients and nursing staff. *Int J Ment Health Nurs* 2020;29:427-439.

19 Puzzo I, Aldridge-Waddon L, Bush E, et al. The relationship between ward social climate, ward sense of community, and incidents of disruptive behaviour: a study of a high secure psychiatric sample. *Int J Forensic Ment Health* 2019;18:153-163.

20 Nijman H L I. A model of aggression in psychiatric hospitals. *Acta Psychiatr Scand* 2002;106:142-143.

21 National Institute for Health and Care Excellence. Violence and aggression: short-term management in mental health, health and community settings. 2015. Available at: https://www.nice.org.uk/guidance/ng10 (Accessed 31 August 2020)

22 Creek, J. Approaches to practice. In: Bryant W, Fieldhouse J, Bannigan K, eds. Creek's occupational therapy and mental health. London: Churchill Livingstone Elsevier 2014: 50-71.

23 Creek J. The core concepts of occupational therapy practice: a dynamic framework for practice. London: Jessica Kingsley Publishers 2010.

24 de Vogel V, de Ruiter C, Bouman Y, et al. SAPROF: guidelines for the assessment of protective factors for violence risk. English version. Utrecht: Forum Educatief 2007.

25 Blank A. Patient violence in community mental health: a review of literature. *Br J Occup Ther* 2001;64:584-859.

26 Kielhofner G. Model of human occupation: theory and application. 4th edn. Baltimore: Lippincott, Williams and Wilkins 2008.

27 Wilcock A. Reflections on doing, being and becoming. *Aust Occup Ther J* 1999;46:1-11.

28 Twinley R. The dark side of occupation: a concept for consideration. *Aust Occup Ther J* 2012;60:301-303.

29 Twinley R, and Addidle G. Considering violence: the dark side of occupation. *Br J Occup Ther* 2012;75:202-204.

30 Aldrich R M, and White N. Reconsidering violence: a response to Twinley and Addidle (2012) and Morris (2012). *Br J Occup Ther* 2012;75:527-529.

31 Law M, Cooper B, Strong S, et al. The person-environment-occupation model: a transactive approach to occupational performance. *Can J Occup Ther* 1996;63:9-23.

32 Rai S. Preventing workplace aggression and violence: a role for occupational therapy. *Work* 2002;18:15-22.

For peer review only - http://bmjopen.bmj.com/site/about/guidelines.xhtml

33 Robbins J E. Violence prevention in schools: implications for occupational therapy. *Work* 2001;17:75-82.

34 Arksey H, and O'Malley L. Scoping studies: towards a methodological framework. *Int J Soc Res Methodol* 2005;8:19-32.

35 Peters M D J, Godfrey C, McInerney P, et al. Chapter 11: Scoping Reviews. In: Aromataris E, and Munn Z, eds. Joanna Briggs Institute Reviewer's Manual. 2020. Available at: https://reviewersmanual.joannabriggs.org (Accessed 13 June 2020).

36 Levac D, Colquhoun H, and O'Brien K K. Scoping studies: advancing the methodology. *Implementation Science* 2010;5:69-77.

37 Tricco A C, Lillie E, Zarin W, et al. PRISMA Extension for scoping reviews (PRISMA-ScR) checklist and explanation. *Ann Intern Med* 2018;169:467-473.

38 Tomlin G, and Borgetto B. Research pyramid: a new evidence-based practice model for occupational therapy. *Am J Occup Ther* 2011;65:189-196.

39 Ouzzani M, Hammady H, Fedorowicz Z, et al. Rayyan: a web and mobile app for systematic reviews. *Syst Rev* 2016;5:210-219.

40 Khalil H, Peters M, Godfrey C M, et al. An evidence-based approach for scoping reviews. *Worldviews Evid Based Nurs* 2016;13:118-123.

41 Cooper S, Cant R, Kelly M, et al. An evidence-based checklist for improving scoping review quality. *Clin Nurs Res* 2019:1-24.

42 Daudt H M, van Mossel C, and Scott S J. Enhancing the scoping study methodology: a large, inter-professional team's experience with Arksey and O'Malley's framework. *BMC Med Res Methodol* 2013;13:48-56.

43 Verhage A, and Boels, D. Critical appraisal of mixed methods research studies in a systematic scoping review on plural policing: assessing the impact of excluding inadequately reported studies by means of a sensitivity analysis. *Qual Quant* 2017:51;1449-1468.

44 Carroll C, Booth A, and Cooper K. A worked example of 'best-fit' framework synthesis: a systematic review of views concerning the taking of some potential chemopreventative agents. *BMC Med Res Methodol* 2011;11:21-29.

45 Braun B, and Clarke V. Using thematic analysis in psychology. *Qual Res Psychol* 2006;3:77-101.

For peer review only - http://bmjopen.bmj.com/site/about/guidelines.xhtml

Appendix One – Literature Search Strategy for CINAHL Plus

Search	Date	Query	Result
S4	05/06/2021	S1 and S2 and S3	184
S3	05/06/2021	occupational therap* OR occupational scien* OR occupational performance OR occupational participation OR activity analysis OR occupational identity OR occupational alienation OR occupational deprivation OR occupational disruption OR occupational dysfunction OR occupational imbalance OR occupational adaptation OR occupational *justice OR occupational engagement OR occupationa* focus* OR occupational perspective OR occupational profile OR Model of Human Occupation OR MOHO OR Canadian Model of Occupational Performance and Engagement OR CMOP-E OR Person?Environment?Occupation?Performance model OR PEOP OR Vona du Toit Model of Creative Ability OR VdTMoCA OR Kawa OR Person?environment?occupation OR PEO model OR dark occupation OR sensory OR vocational rehabilitation	100,300
S2	05/06/2021	aggressi* OR violen* OR agitat* OR hostil* OR irritab* OR assault* OR seclusion OR restraint OR offending OR recidivism	140,970
S1	05/06/2021	mental health OR psychiatry OR mental illness OR psychiatric illness OR mental disorder OR psychiatric disorder OR psychopath* OR personality disorder	255,519

For peer review only

Preferred Reporting Items for Systematic reviews and Meta-Analyses extension for Scoping Reviews (PRISMA-ScR) Checklist

SECTION	ITEM	PRISMA-ScR CHECKLIST ITEM	REPORTED ON PAGE #
**TITLE**			
Title	1	Identify the report as a scoping review.	1
**ABSTRACT**			
Structured summary	2	Provide a structured summary that includes (as applicable): background, objectives, eligibility criteria, sources of evidence, charting methods, results, and conclusions that relate to the review questions and objectives.	2
**INTRODUCTION**			
Rationale	3	Describe the rationale for the review in the context of what is already known. Explain why the review questions/objectives lend themselves to a scoping review approach.	5-6
Objectives	4	Provide an explicit statement of the questions and objectives being addressed with reference to their key elements (e.g., population or participants, concepts, and context) or other relevant key elements used to conceptualize the review questions and/or objectives.	6
**METHODS**			
Protocol and registration	5	Indicate whether a review protocol exists; state if and where it can be accessed (e.g., a Web address); and if available, provide registration information, including the registration number.	N/A
Eligibility criteria	6	Specify characteristics of the sources of evidence used as eligibility criteria (e.g., years considered, language, and publication status), and provide a rationale.	6-7
Information sources*	7	Describe all information sources in the search (e.g., databases with dates of coverage and contact with authors to identify additional sources), as well as the date the most recent search was executed.	7
Search	8	Present the full electronic search strategy for at least 1 database, including any limits used, such that it could be repeated.	Appendix 1
Selection of sources of evidence†	9	State the process for selecting sources of evidence (i.e., screening and eligibility) included in the scoping review.	7-8
Data charting process‡	10	Describe the methods of charting data from the included sources of evidence (e.g., calibrated forms or forms that have been tested by the team before their use, and whether data charting was done independently or in duplicate) and any processes for obtaining and confirming data from investigators.	8
Data items	11	List and define all variables for which data were sought and any assumptions and simplifications made.	6-7
Critical appraisal of individual sources of evidence§	12	If done, provide a rationale for conducting a critical appraisal of included sources of evidence; describe the methods used and how this information was used in any data synthesis (if appropriate).	8

For peer review only - http://bmjopen.bmj.com/site/about/guidelines.xhtml

SECTION	ITEM	PRISMA-ScR CHECKLIST ITEM	REPORTED ON PAGE #
Synthesis of results	13	Describe the methods of handling and summarizing the data that were charted.	8-9
**RESULTS**			
Selection of sources of evidence	14	Give numbers of sources of evidence screened, assessed for eligibility, and included in the review, with reasons for exclusions at each stage, ideally using a flow diagram.	N/A
Characteristics of sources of evidence	15	For each source of evidence, present characteristics for which data were charted and provide the citations.	N/A
Critical appraisal within sources of evidence	16	If done, present data on critical appraisal of included sources of evidence (see item 12).	N/A
Results of individual sources of evidence	17	For each included source of evidence, present the relevant data that were charted that relate to the review questions and objectives.	N/A
Synthesis of results	18	Summarize and/or present the charting results as they relate to the review questions and objectives.	N/A
**DISCUSSION**			
Summary of evidence	19	Summarize the main results (including an overview of concepts, themes, and types of evidence available), link to the review questions and objectives, and consider the relevance to key groups.	N/A
Limitations	20	Discuss the limitations of the scoping review process.	N/A
Conclusions	21	Provide a general interpretation of the results with respect to the review questions and objectives, as well as potential implications and/or next steps.	N/A
**FUNDING**			
Funding	22	Describe sources of funding for the included sources of evidence, as well as sources of funding for the scoping review. Describe the role of the funders of the scoping review.	12

JBI = Joanna Briggs Institute; PRISMA-ScR = Preferred Reporting Items for Systematic reviews and Meta-Analyses extension for Scoping Reviews.

* Where *sources of evidence* (see second footnote) are compiled from, such as bibliographic databases, social media platforms, and Web sites.

† A more inclusive/heterogeneous term used to account for the different types of evidence or data sources (e.g., quantitative and/or qualitative research, expert opinion, and policy documents) that may be eligible in a scoping review as opposed to only studies. This is not to be confused with *information sources* (see first footnote).

‡ The frameworks by Arksey and O'Malley (6) and Levac and colleagues (7) and the JBI guidance (4, 5) refer to the process of data extraction in a scoping review as data charting.

§ The process of systematically examining research evidence to assess its validity, results, and relevance before using it to inform a decision. This term is used for items 12 and 19 instead of "risk of bias" (which is more applicable to systematic reviews of interventions) to include and acknowledge the various sources of evidence that may be used in a scoping review (e.g., quantitative and/or qualitative research, expert opinion, and policy document).

From: Tricco AC, Lillie E, Zarin W, O'Brien KK, Colquhoun H, Levac D, et al. PRISMA Extension for Scoping Reviews (PRISMAScR): Checklist and Explanation. Ann Intern Med. 2018;169:467–473. doi: 10.7326/M18-0850.

For peer review only - http://bmjopen.bmj.com/site/about/guidelines.xhtml
